# Identification of Kaurane-Type Diterpenes as Inhibitors of Leishmania Pteridine Reductase I

**DOI:** 10.3390/molecules26113076

**Published:** 2021-05-21

**Authors:** Chonny Herrera-Acevedo, Areli Flores-Gaspar, Luciana Scotti, Francisco Jaime Bezerra Mendonça-Junior, Marcus Tullius Scotti, Ericsson Coy-Barrera

**Affiliations:** 1 Post-Graduate Program in Natural and Synthetic Bioactive Products, Federal University of Paraíba, João Pessoa 58051-900, PB, Brazil; chonny622@gmail.com (C.H.-A.); luciana.scotti@gmail.com (L.S.); 2 Bioorganic Chemistry Laboratory, Facultad de Ciencias Básicas y Aplicadas, Universidad Militar Nueva Granada, Cajicá 250247, Colombia; ericsson.coy@unimilitar.edu.co; 3 Departamento de Química, Facultad de Ciencias Básicas y Aplicadas, Universidad Militar Nueva Granada, Cajicá 250247, Colombia; 4 Laboratory of Synthesis and Drug Delivery, State University of Paraíba, João Pessoa 58071-160, PB, Brazil; franciscojbmendonca@yahoo.com.br

**Keywords:** *Leishmania*, natural products, kauranes, Asteraceae, virtual screening, machine learning, molecular docking

## Abstract

The current treatments against *Leishmania* parasites present high toxicity and multiple side effects, which makes the control and elimination of leishmaniasis challenging. Natural products constitute an interesting and diverse chemical space for the identification of new antileishmanial drugs. To identify new drug options, an *in-house* database of 360 kauranes (tetracyclic diterpenes) was generated, and a combined ligand- and structure-based virtual screening (VS) approach was performed to select potential inhibitors of *Leishmania major* (*Lm*) pteridine reductase I (PTR1). The best-ranked kauranes were employed to verify the validity of the VS approach through *Lm*PTR1 enzyme inhibition assay. The half-maximal inhibitory concentration (IC_50_) values of selected bioactive compounds were examined using the random forest (RF) model (i.e., 2β-hydroxy-menth-6-en-5β-yl *ent*-kaurenoate (**135**) and 3α-cinnamoyloxy-*ent*-kaur-16-en-19-oic acid (**302**)) were below 10 μM. A compound similar to **302**, 3α-*p*-coumaroyloxy-*ent*-kaur-16-en-19-oic acid (**302a**), was also synthesized and showed the highest activity against *Lm*PTR1. Finally, molecular docking calculations and molecular dynamics simulations were performed for the VS-selected, most-active kauranes within the active sites of PTR1 hybrid models, generated from three *Leishmania* species that are known to cause cutaneous leishmaniasis in the new world (i.e., *L. braziliensis*, *L. panamensis,* and *L. amazonensis*) to explore the targeting potential of these kauranes to other species-dependent variants of this enzyme.

## 1. Introduction

Leishmaniasis refers to a group of anthroponotic and zoonotic diseases that affect between 700,000 and 1 million people worldwide, causing between 20,000 and 30,000 deaths each year, primarily among populations found in tropical and subtropical areas. Leishmaniasis has been classified as a neglected tropical disease (NTD) due to lack of research and the poor development of new drugs over many decades [1,2,3]. Leishmaniasis is caused by approximately 20 protozoan parasite species of the genus *Leishmania*, which are transmitted to humans by more than 30 different species of phlebotomine sandflies [4]. The distinct species of *Leishmania* cause at least four separate syndromes: visceral leishmaniasis (VL, also known as kala-azar), post-kala-azar dermal leishmaniasis (PKDL), cutaneous leishmaniasis (CL), and mucocutaneous leishmaniasis (MCL) [5].

The CL subtype is typically characterized by localized, diffuse, or disseminated skin lesion [6]. In the old world (southern Europe, the Middle East, southwest Asia, and Africa), approximately 20 different *Leishmania* species are responsible for the transmission of CL, including *L tropica, L. major, L. aethiopica, L. infantum,* and *L. donovani*, with *L. major* representing the most common causative organism [7]. In the new world (from the southern United States through Latin America to South America), *L. mexicana*, *L. venezuelensis*, *L. amazonensis*, *L. braziliensis*, *L. panamensis*, *L. guyanensis,* and *L. peruviana* are the primary causal species of CL [8,9]. In Colombia, the overall leishmaniasis incident rate is 26.2 cases per 100,000 population (including 98.6% of the cases related to CL), and in Brazil, autochthonous cases of CL have been reported in all states. Colombia and Brazil represent the new world countries with the most frequently reported CL clinical manifestation [10,11].

Starting in the 1950s, pentavalent antimonial compounds were introduced as treatments against *Leishmania* species; however, these drugs are associated with several adverse events and are becoming increasingly ineffective due to the development of resistance [12,13]. Other drugs used to treat leishmaniasis include amphotericin B in a liposomal formulation, which significantly reduced the side effects and treatment duration associated with amphotericin B in the free form but is very expensive; and paromomycin and miltefosine, which are associated with high toxicity (particularly renal toxicity), increased resistance, and teratogenic and abortifacient effects [4,12]. Therefore, alternative chemotherapies must be developed to improve the control and elimination of this group of diseases. Natural products, which have always been an important source of bioactive compounds, are commonly used as the starting material for new drug development [14,15,16].

Computational studies using natural products have been reported in the continuous search for new leishmanicidal drugs or lead compounds. In particular, machine learning and molecular docking calculations have been used to identify new structures with potential anti-*Leishmania* activities, based on secondary metabolites found in Asteraceae species [17,18], especially sesquiterpenoids [19,20], triterpenes [21], and phytosterols [22].

Specifically, *ent*-kauranes are an important group of tetracyclic diterpenes, and their structures are constituted by a perhydrophenantrene unit fused with a cyclopentane unit formed by a bridge of two carbons between C-8 and C-13 [23]. Despite more than 1300 *ent*-kaurane diterpenoids have been isolated and identified from different plant sources, and a wide range of biological activities being reported (i.e., anti-inflammatory, antibacterial and anticancer), anti-*Leishmania* studies examining the effects of *ent*-kauranes, a common class of secondary metabolites found in Asteraceae are rare [24,25,26]. Nogueira et al., found that ent-3-α-hydroxy-kaur-16-en-18-ol showed significant in vitro activity against both *Plasmodium falciparum* (IC_50_ = 3.5 μM) and *Leishmania donovani* (IC_50_ = 2.5 μM) with high selectivity indices (Selectivity index, SI > 10) in comparison with L6 cells [27]. Orduz-Diaz evaluated thirteen known kaurane-related diterpenes against *Leishmania major* PTR1 by molecular docking and pharmacophore modeling, showing that kauren-19-oic acids possess significant structural features for inhibition of PTR1 [28].

Thus, in this study, an in silico approach, combining both structure- and ligand-based virtual screening (VS), was used to select structures with potential activity against pteridine reductase 1 (PTR1) from *L. major* (*Lm*PTR1*)* from an in-house database containing 360 kauranes. PTR1 (E.C. 1.5.1.33), an NADPH-dependent short-chain reductase, is responsible for the unusual salvage of pterin in *Leishmania* and acts as a metabolic bypass for drugs that target dihydrofolate reductase [29].

Subsequently, the in silico results were verified through in vitro assays, determining the half-maximal inhibitory concentrations (IC_50_) for the structures **135**, **301,** and **302**. In addition, two derivatives structures (**301a** and **302a**) were synthesized, and their IC_50_ values were also calculated. Finally, molecular docking and molecular dynamics simulations were performed to identify potential kauranes against PTR1 of various *Leishmania* species known to cause CL in the new world.

## 2. Results and Discussion

### 2.1. A Combined Ligand-/Structure-Based Virtual Screening Approach Using LmPTR1

#### 2.1.1. Ligand-Based VS

The ChEMBL dataset (https://www.ebi.ac.uk/chembl/, accessed on 10 January 2021) was classified as either active or inactive (binary classification), using a cutoff value of pIC_50_ ≥ 5.0 (pIC_50_ = log IC_50_). This value was selected according to the range of pIC_50_ values observed for the entire dataset (657 structures) to obtain the maximum representation of the chemical space for each class of structure (active and inactive). Structures with pIC_50_ values between 4.9 and 5.0 (range of 0.1 units) were excluded to avoid edge effects and improve the predictive capacity of the models by minimizing the potential activity differences associated with errors and different experimental protocols. IC_50_ values describe the concentration of a given substance required to inhibit 50% of parasite growth [20].

Subsequently, VolSurf+ descriptors (128) were calculated for the remaining molecules, including 298 inactive (46.9%) and 338 active (53.1%) molecules. All VolSurf+ descriptors [30,31] together with their respective binary classifications were used to build a random forest (RF) model in Knime software (KNIME 3.1.0 the Konstanz Information Miner Copyright, 2003–2014, www.knime.org) [32]. A model with 200 trees was selected, and the Gini Index was used as a split criterion, which has the lowest false-positive rate. A five-fold cross-validation procedure was performed, splitting the dataset five times into a modeling set (80%/20%). Only the modeling set, which was additionally divided into multiple training and test sets (80%/20%), was used to build and validate the models [33].

For the training set used in the RF model, the match percentage values approached 100%. Sensitivity (true-positive rate) values of 78.1% and 82.6% and specificity (true-negative rate) values of 72.7% and 73.7%, were obtained for the cross-validation and test sets, respectively. Two parameters were calculated to evaluate the quality of the RF model: the receiver operating characteristic (ROC) curve and Matthews’s correlation coefficient (MCC). The area under the ROC curve (AUC) plots the true-positive rate (sensitivity) against the false-positive rate (1−specificity), and the MCC correlates all values in the confusion matrix [34,35].

For the *L. major* RF model, AUC values of 0.85 and 0.87 were obtained for the five-fold cross-validation and external validation datasets, respectively. When calculating the MCC parameter, a value of 1 represents a perfect prediction, a value of 0 represents a random prediction, and a value of −1 represents total disagreement between the prediction and the observation. Our *L. major* RF model returned values of 0.51 (five-fold cross-validation) and 0.57 (external validation) [35]. The slightly higher MCC value obtained for the external validation (0.57) demonstrates a high degree of differentiation between the active and inactive compounds identified in the ChEMBL dataset.

The applicability domain (APD) was used to assess the reliability of the predictions for the samples in the test and SL sets, and the calculation of the APD is based on the molecular interactions determined by the VolSurf+ descriptors [14,20]. For the *L. major* RF model set, more than 98.4% of molecules were classified as reliable, with only 8 molecules classified as unreliable. When the RF models were applied to the kaurane dataset, more than 94.2% of molecules were classified as reliable in each model, with only 20 molecules classified as unreliable. Unreliable molecules were removed.

**Figure 1:**
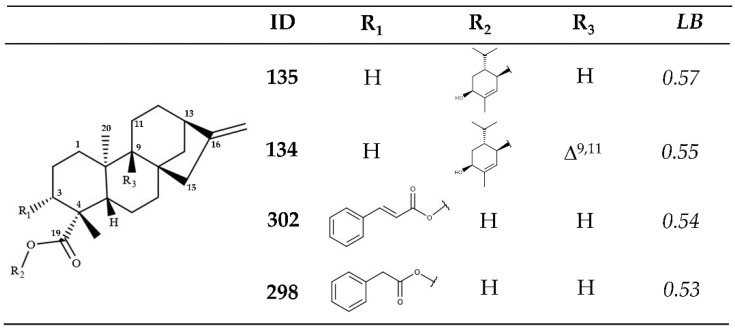
Potentially active kauranes, identified using RF model (ligand-based VS), for *L. major*. *LB* active probability value.

A ligand-based VS was performed on the remaining 340 kauranes. Only 7 of the 340 structures were classified as active (ligand-based probability value [*LB*] ≥ 0.5), with structures **134** and **135** representing the two best-ranked kauranes, with *LB* values of 0.57 and 0.55, respectively (Figure 1). These two diterpenoids are found in *Wedelia chinensis*, a species of Asteraceae [36]. Structurally, these two kauranes are characterized by the presence of (1*S*,4*R*,5*R*)-2-methyl-5-propan-2-ylcyclohex-2-ene-1,4-diol, linked through an ester bond to the kaurenoic acid. The *LB* values for these two kauranes are almost identical, indicating that the activity of these two compounds is likely associated with the presence of this monoterpenoid and the pi-bond in the structure between C9 and C11.

Additionally, two additional kauranes isolated from *Wedelia trilobata*, structures **298** and **302,** also presented *LB* values greater than 0.5. Cinnamoyl (**302**) and 2-phenylacetic (**298**) esters are established with the carboxyl group of the kaurenoic acid (Figure 1) [37]. In these two structures, the functional groups present in R3 were also found to play key roles, as structure **301**, which also includes a cinnamoyl ester, was classified as inactive (*LB* = 0.48). The presence of a hydroxyl moiety in R3 represents the unique structural difference between structures **301** and the active structure **302**.

#### 2.1.2. Structure-Based VS

A structure-based VS (molecular docking) was performed to explore the mechanism of action of the kauranes dataset against the crystal structure of PTR1 (E.C. 1.5.1.33), an NADPH-dependent short-chain reductase that is responsible for the unusual salvage of pterin in *Leishmania* and acts as a metabolic bypass for drugs that target dihydrofolate reductase [29]. The docking scores and the respective deviation values for the best-ranked structures are reported in Table 1 (all binding energy values can be found in Supplementary Material, Table S3). All tested molecules were ranked using the following probability calculation (Equation (1)), as previously reported by Herrera-Acevedo et al. [14,20]. Those kauranes that presented structure-based probability values (*SB*) above 0.5 were classified as active.

*SB* = (*E_i_*/*E_min_*) > 0.5 and *E_i_* < *E_ligand_* (1)

where *SB* is the structure-based probability; *E_i_* is the docking energy of compound *i*, where *i* ranges from 1 to 360 (Kauranes dataset); *E_min_* is the lowest energy value of the dataset; and *E_ligand_* is the ligand energy from protein crystallography.

The docking results showed that all 360 compounds obtained *SB* values above 0.5; however, relative to the PTR1 inhibitors that were used as controls, 252 structures and 359 structures had lower docking scores than 7,8-dihydro-l-biopterin (DHB) and pyrimethamine (PMA), respectively. The Protein Data Bank (PDB) ligand for *Lm*PTR1, methotrexate (PDB ID: MTX) [38], has a calculated docking score of −560.4 kJ/mol.

**Figure 2:**
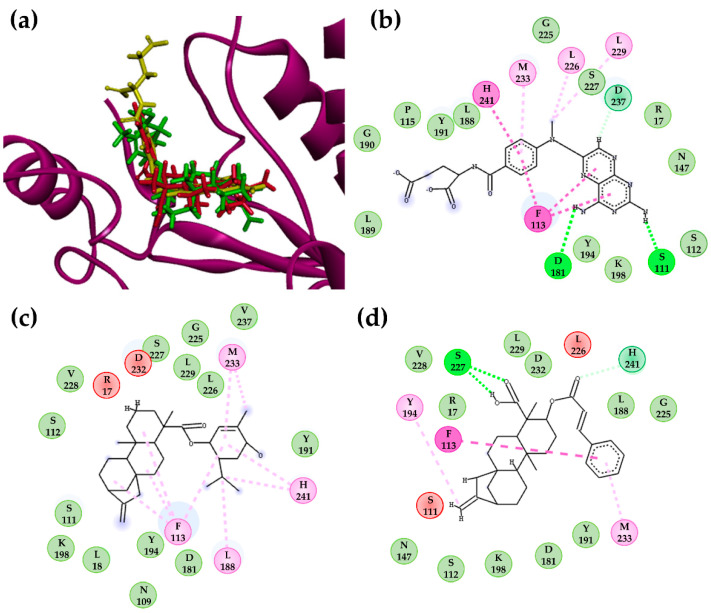
(**a**) Docking conformations of structure **135** (green)**, 302** (red) and MTX (yellow) in the active site of *Lm*PTR1; 2D-residual interaction diagrams of (**b**) methotrexate (MTX), (**c**) structure **135,** and (**d**) structure **302**. Interacting residues are shown as colored circles depending on the interactions (as colored dashed lines): H-bond (lime), Van der Waals (green), π–π (purple) and π–alkyl (pink), unfavorable (red), and carbon H-bond (teal) interactions.

Structures **135** and **302** (Figure 1), which were predicted to have high *LB* probability values based on the RF model, also showed high *SB* values and were two of the ten best-ranked kauranes identified, with *SB* values of −423.0 kJ/mol and −416.7 kJ/mol, respectively. Spatially, in the active site of *Lm*PTR1, structures **135** and **302** adopted an *L-shaped* conformation, similar to that observed for the ligand methotrexate (Figure 2a). Based on the two-dimensional analysis, common interactions were identified for these two kauranes compared with methotrexate, highlighting the π-alkyl interaction with M233 and the van der Waals interactions with S112, Y191, K198, and G225 (Figure 2).

Methotrexate achieved a docking score of −560.4 kJ/mol in the active site of *Lm*PTR1, and the formation of two H-bond interactions with S111 and N118 were observed (Figure 2b). Structure **302** also formed two H-bonds between S227 and the carboxylic group of C-19. Additionally, the aromatic ring of F113 interacted with both **135** and **302**, in addition to methotrexate, through π–π and π–alkyl interactions. Two steric interactions that unfavorably influenced the molecular binding energy were identified for the structures **135** (R17 and D232) and **302** (S111 and L226), as shown in Figure 2c,d, respectively.

#### 2.1.3. Consensus Analysis of the Two VS Approaches

To verify the potentially active kauranes and their possible mechanisms of action, a combined approach using both structure- and ligand-based VS was performed. An equation was used to combine the probability scores of both VS approaches with the true-negative rate from the RF model to minimize the probability of selecting false-positive compounds (Equation (2)) [14,20].

*CA_Lm_* = [*SB* + (1 + *TN*) × *LB*]/[2 + *TN*] (2)

where *CA_Lm_* = combined-approach probability, *SB* = structure-based probability, *TN* = true-negative rate, and *LB* = ligand-based probability.

Equation (2) is based on the fact that the ligand-based VS uses pIC_50_ experimental values; thus, the *LB* score has a high weight with respect to the *SB* score, which only relates interactions between the protein and ligand. The ligand-based VS seeks to reduce the probability of selecting inactive molecules as active compounds (false positive); therefore, in Equation (2), the *LB* is associated with an increment of TN.

Table 2 shows the results for the five kauranes that were classified as active using the combined approach and the two VS methodologies. Four of the five structures that previously displayed a high active probability value in the ligand-based VS (Figure 1) emerged as interesting potential anti-*Leishmania* structures that might act on the *Lm*PTR1 protein.

In addition, fischericin F (structure **101**), extracted from *Ligularia fischeri,* a species of the *Ligularia* genus (Asteraceae) [39], was also classified as potentially active in the combined approach (*CA_Lm_* = 0.69). Although this kaurane did not present the highest scores from the ligand-based VS, it emerged as the best-ranked structure from the structure-based VS approach. Structurally, 101 has ferulic acid as the main feature, bound to the *ent*-kaurane skeleton through an ester bond at C14.

Through this combined approach, based on two different VS methodologies, five kauranes from various Asteraceae species were identified as having promising antileishmanial activity against *Lm*PTR1 from a dataset of 360 kauranes, with structures 302 and 135 indicated as having high probability values based on both the ligand-based and structure-based VS approaches.

### 2.2. In Vitro Enzymatic Activity Inhibition for VS-Selected Kauranes against Lmptr1

To verify the results obtained from the combined approach using the two VS methodologies, the in vitro enzymatic inhibitory activities of structures **135** and **302** (actives) and structure **301** (inactive) were examined. In addition, two kauranes, structures **301a** and **302a** (in which the cinnamoyloxy group was replaced by a coumaroyloxy group), were also tested against *Lm*PTR1. The diterpenes **135**, **301**, and **302** were synthesized for use in an in vitro enzymatic activity inhibition assay. This aim was oriented to identify appropriate precursors, as such compounds are not commercially available. Therefore, a phytochemical study was initially performed focusing on the fruits of *Xylopia frutescens*, an annonaceous plant that is rich in kaurane-type diterpenes [40].

**Figure 3:**
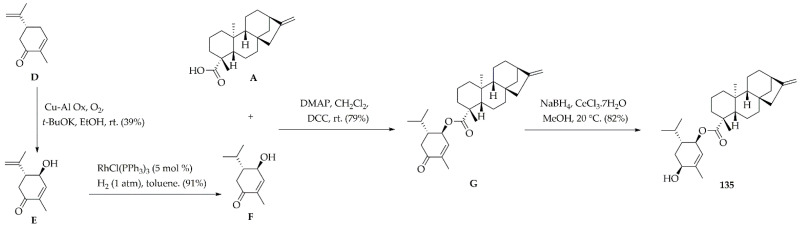
Synthetic route to produce monoterpene/diterpene ester adduct **135**.

This procedure led to the isolation of various diterpenes, but our interest was particularly focused on *ent*-kaurenoic acid (**A**), 3α-hydroxy-*ent-*kaur-16-en-19-oic acid (**B**), and 3α,9β-dihydroxy-*ent-*kaur-16-en-19-oic acid (**C**). The structures of these compounds were fully elucidated by detailed spectroscopic data interpretation and comparison with data available in the literature for compounds **A** and **B** [41,42]. Compound **C** has not been yet reported, but it was found to have very similar ^1^H and ^13^C NMR data to that of the previously reported pterokaurene L3 (PL3) [43]. This fact suggested that **C** and PL3 are structural analogues, whose spectral difference was solely found to be on the chemical shift at C-3 (δ_C_ 78.6 in **C** vs. 38.6 in PL3), confirming the presence of a carbinol carbon having an α-oriented hydroxyl group. This α-orientation was deduced by the ^1^H NMR data of H-3 (δ_H_ 4.63, dd, 1H), specifically the consistent coupling constants between H-3β and H-2α (*J* = 12 Hz) and H-2β (*J* = 5 Hz) [37]. These kaurane-type diterpenes **A**–**C** were therefore considered suitable precursors to initiate the synthesis of target compounds. Thus, compound 135 was obtained from the commercially available (*R*)-(−)-carvone (**D**) (Figure 3), which was first transformed into 5β-hydroxy-(*R*)-carvone (**E**) by chemoselective monohydroxylation and subsequently reduced to 2-oxo-menth-6-en-5β-ol (**F**) by selective hydrogenation using the Wilkinson’s catalyst [44].

Diterpenic acid A esterified with F under mild conditions via Steglich esterification [45] to produce 2-oxo-menth-6-en-5β-yl *ent*-kaurenoate (**G**). This monoterpene/diterpene ester adduct was finally converted into 2β-hydroxy-menth-6-en-5β-yl *ent*-kaurenoate (**135**) through the selective 1,2 reduction of α,β-unsaturated ketones using Luche conditions [46], whose Re face of the enone in **G** favored the desired β-epimer (68% epimeric excess).

**Figure 4:**
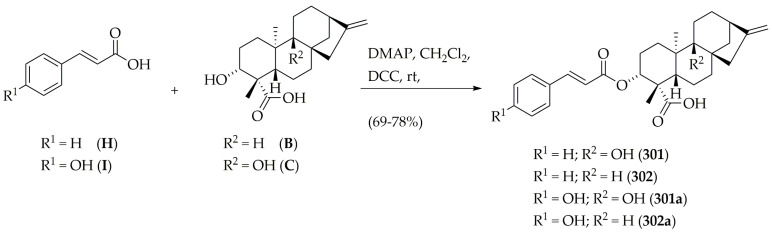
Synthetic route to produce phenylpropanoid/diterpene ester adducts **301**, **302**, **301a**, and **302a**.

Steglich esterification was also exploited to obtain the other two selected diterpenes (Figure 4). Isolated compounds **B** and **C** were separately esterified with cinnamic acid (**H**), yielding the phenylpropanoid/diterpene ester adducts **301** and **302**, respectively, with good yields (78%–79%). Additionally, the scope of this reaction was expanded to produce compounds **301a** and **302a**, using the same diterpene precursors (**B** and **C**) condensed with *p*-coumaric acid (I) to observe the influence of the *p*-hydroxyl group at the phenylpropanoid moiety in the subsequent enzymatic study.

The selected synthetic diterpene esters **135**, **301**, **302**, **301a**, and **302a** were tested in vitro to experimentally determine their abilities to inhibit the enzymatic activity of *Lm*PTR1 as an extension of the results provided by the in silico screening. Recombinant *Lm*PTR1 was kinetically assessed, as previously reported [40], to ensure the appropriate enzymatic features, resulting in a consistent substrate Km of 5.6 µM. After testing *Lm*PTR1 inhibition, the selected diterpenes exhibited inhibitory properties at different levels, following a concentration-response behavior within the 0.1–128 µM range. The IC_50_ was then calculated for the tested diterpenes, and these values were used to calculate the apparent inhibitory constant (Ki^app^) (Table 3) using the Cheng–Prusoff equation, assuming reversible competitive inhibition and 1:1 stoichiometry [47]. PMA, a known PTR1 inhibitor, was used as a positive control. Among the three VS-selected diterpenes, **135** was found to be the most potent inhibitor, whereas **301** exhibited the lowest Ki^app^. Remarkably, the inhibitory activity was improved by approximately 60% if a 3α-*p*-coumaroyloxy group was present in **302** instead of a 3α-cinnamoyloxy substituent, as **302a** exhibited a lower Ki^app^ value than **302**. No similar effect was observed for **301**, as **301a** showed a slightly lower inhibitory activity than 301. Therefore, a reasonable inference based on this small set of compounds is that the presence of a *p*-hydroxyl group at the phenylpropanoid moiety might favor inhibitory activity, whereas a 9β-hydroxyl group at the diterpene moiety has a negative influence on *Lm*PTR1 inhibition.

Finally, although the test diterpenes were found to be less active than the positive control, the concentration-response behavior and the consequently calculated Ki^app^ (≤5 µM) of the selected diterpenes demonstrated the validity of the designed VS approach for the selection of bioactive compounds against PTR1 and the computationally studied binding modes of these selected compounds within the active site of *Lm*PTR1, which is associated with the development of CL. These selected compounds can be considered important, showing that they can be used to obtained additional active PTR1 inhibitors.

### 2.3. Molecular Docking Calculations for the Kaurane Dataset Using Hybrid Models of La, Lb, and Lpptr1

The structures **135**, **302**, and **302a** displayed in vitro activity against *L. major,* which is one of the species responsible for most CL cases in the Mediterranean littoral, the Middle East, the Indian subcontinent, and central Asia [48]. However, in the American continent, other *Leishmania* species, such as *L. amazonensis (La)*, *L. braziliensis (Lb)*, and *L. panamensis (Lp),* are associated with great clinical diversity, associated particularly with CL and MCL [49]. Therefore, the potential activity of kauranes against PTR1 from these three species must also be examined, despite the absence of crystal structure for these species.

#### 2.3.1. Hybrid Models of *La*, *Lb*, and *Lp*PTR1

Hybrid models were built in YASARA software (YASARA (18.4.24) Vienna, Austria: YASARA Biosciences GmbH; 2018) [50] from sequences of three *Leishmania* species, *Lp*, *La,* and *Lb.* To verify and validate the reliability and stereochemical qualities of the modeled protein, data from Ramachandran, WHAT IF, and VERIFY 3D plots and the quality Z-scores of dihedrals were determined for the built models, which describes how many standard deviations separate the model quality from the average high-resolution X-ray structure [51]. Higher values are better, and negative values indicate that the homology model looks worse than a high-resolution X-ray structure [52,53]. The Ramachandran plot showed that the main possible chain conformations included more than 88.7% of residues in the most favored regions for the three hybrid models, with close to 10.0% of residues in allowed regions. Only the *Lp* model showed two residues (0.8%) in disallowed regions (outliers; Supplementary Material, Figure S2). Because the percentage of residues found in the outlier region was low or absent, the generated models were considered satisfactory. Eleven residues in the active site were analyzed against the template sequence and were found to be conserved [38].

According to the VERIFY 3D results (https://services.mbi.ucla.edu/SAVES/, accessed on 13 February 2021), 87.1% (*Lp*), 86.1% (*Lb*), and 80.0% (*La*) of residues had mean 3D/1D scores ≥ 0.2, which indicated a reliable model because more than 80% of amino acids had values of 0.2 in the 3D/1D profile. The verification of dihedral quality was classified as optimal for the three models, with values above 0.913. The quality of atomic contacts between the atoms of each residue was analyzed using the Coarse Packing Quality Control module of WHAT IF (https://swift.cmbi.ru.nl/servers/html/index.html, accessed on 15 February 2021), which compares the distribution of atom positions around each residue. The mean scores of all wastes were −0.334*,* −0.488, and −0.667, for *Lb*, *La,* and *Lp,* respectively. A score of less than −5.0 for a residue indicates poor or unusual atomic contacts.

#### 2.3.2. Molecular Docking Calculations for Kauranes Dataset

Molecular docking calculations for the 360 kaurane dataset plus the two derivative compounds, **301a** and **302a**, were obtained using the Autodock/Vina algorithm for the three generated *Leishmania* hybrid models (*Lp*, *Lb,* and *La*) to evaluate whether the kauranes that showed in vitro activity against *L. major* have the potential to display multispecies activity. Equation (3) combines the *SB* probability scores obtained from the docking calculations of all three models, and DHB and PMA were used as references.

*CA* = [(*LaSB* + *LbSB* + *LpSB*)/3] ≥ 0.5 (3)

where *LbSB* is the structure-based probability score for *L. braziliensis*, *LpSB* is the structure-based probability score for *L. panamensis,* and *LaSB* is the structure-based probability score for *L. amazonensis*. *CA* is the consensus analysis for all three species.

Therefore, a *CA* value equal to or greater than 0.5 is classified as active. Among the 362 structures tested, only 274 were classified as active, and **301a** and **302a** were the best-ranked molecules, with *CA* values of 0.96 and 0.94, respectively. The kauranes (**135** and **302**) that demonstrated in vitro activity against *L. major* also showed high *CA* values (above 0.86) and were among the ten best-ranked molecules (Table 4).

DHB showed more affinity for PTR1 from the three *Leishmania* species than PMA. Lower docking scores than the two control structures were obtained for 100%, 81%, and 99% of the tested kauranes for *Lb, Lp,* and *La,* respectively.

**Figure 5:**
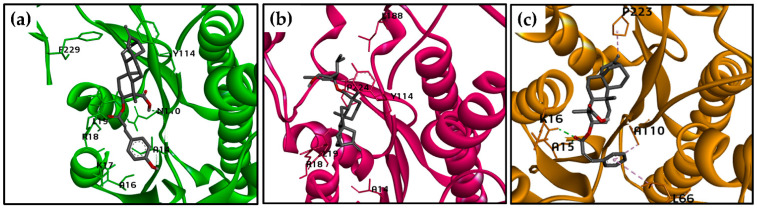
Docking formations between a structure **302** in the active site of (**a**) *Lp*PTR1, (**b**) *Lb*PTR1, and (**c**) *La*PTR1. Labels: H-bonds (green), π-alkyl interactions (purple).

Docking poses for structure **302** in the active site of the three *Leishmania* PTR1 models and the interacting residues for **302**, DHB, and PMA are displayed in Figure 5 and Table 5, respectively. A Vina score of −9.73 kcal/mol was calculated for *Lp*, predominantly due to van der Waals interactions, with five common interactions identified between DHB and PMA (L19, S112, Y194, L226, and S227). A critical common π-anion interaction was observed between D181 and the aromatic ring of the cinnamoyl group. No H-bond interactions are observed for this kaurane in the active site of *Lp*PTR1.

Similarly, the structure of **302** achieved a Vina score of −11.1 kcal/mol in the active site of L*b*PTR1, exhibiting some common van der Waals interactions with DHB and PMA (S112, S227, and L228). An H-bond interaction was established between G225 and the carboxylic group of C-19. G225 did not interact with DHB and PMA, which establish three H-bonds (L19 and N110 were common between these two molecules). Interestingly, an alkyl interaction with L19 was observed for the structures **302** and **135**, which was the best-ranked molecule for *Lb*PTR1 (Vina score of −13.07 kcal/mol).

For structure **302**, in the active site of *La*PTR1, two H-bond interactions were observed with A15 and K16, and K16 was also observed in the complex between *La*PTR1 and DHB, identified as a critical amino acid for the binding. For both the kaurene **302** and the two controls (PMA and DHB), a higher number of van der Waals interactions were exhibited than any other type of intermolecular interaction, although only the interaction with Y193 was common among all three of these structures. Finally, an alkyl interaction with P223 was identified for the structures **302** and PMA.

### 2.4. Molecular Dynamics Simulations

*L. braziliensis* is the causative agent of human CL and MCL in various countries of the American continent, including Colombia, Brazil, Nicaragua, and Ecuador, among others [49,54,55]. Thus, to validate the hybrid model constructed for *Lb*PTR1 and to evaluate the protein–ligand stabilities of the structures **135**, **302**, and **302a**, molecular dynamics (MD) studies were performed using DHB and PMA as reference ligands.

**Figure 6:**
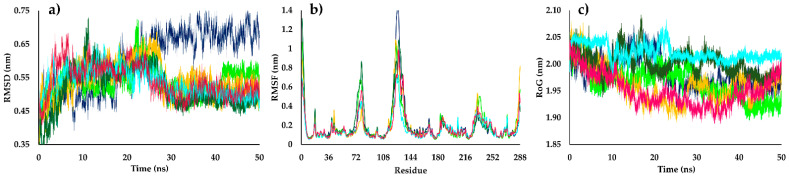
(**a**) Root-mean-square deviation (RMSD), (**b**) root-mean-square-fluctuation (RMSF), and (**c**) radius of gyration (RoG) values within the *Lb*PTR1 binding site, obtained after molecular dynamics simulations using three of the best-ranked structures in molecular docking calculations. apo*Lb*PTR1 (blue); DHB: *Lb*PTR1 complex (light green); PMA: *Lb*PTR1 complex (sky blue); structure **135**: *Lb*PTR1 complex (pink); structure **302**: *Lb*PTR1 complex (yellow); structure **302a**: *Lb*PTR1 complex (dark green).

Root-mean-square deviation (RMSD) values were used to evaluate the structural stability of the receptor frame by measuring the distance between different positions (in nm) that a set of atoms exhibited over time [56]. In the first half of the simulation time (0–25 ns), the structures **135**, **302**, **302a**, DHB, PMA, and the apoenzyme of *Lb*PTR1 (apo*Lb*PTR1, protein without ligand) showed a similar grade of perturbation, with RMSD values ranging from approximately 0.35 to 0.65 nm. After 25 ns, all ligands exhibited reduced perturbations relative to that observed for apo*Lb*PTR1, which suggests increased stability exerted by the inhibitors on the complex with *Lb*PTR1. RMSD values for the protein–kaurane complexes of approximately 0.5 nm were observed, except for the reference ligand, DHB, which showed a slightly higher RMSD value (approximately 0.55 nm). In contrast, apo*Lb*PTR1 exhibited values approaching 0.7 nm (Figure 6a).

The fluctuations for each *Lb*PTR1 residue were analyzed by calculating the root-mean-square fluctuation (RMSF) values. Kauranes, DHB, and PMA in complex with *Lb*PTR1 presented lower values than the apoenzyme, and the *Lb*PTR1 loops were identified as the most variable regions. In the sections of *Lb*PTR1 with defined tertiary structures (helical or β-sheets), the fluctuation of residues for both the apoenzyme and the complexes formed with DHB, PMA, and kauranes (**135**, **302**, and **302a**) was less than 0.25 nm. For most of the residues in the active site, the RMSF values decreased when *Lb*PTR1 was in complex with structure **302**.

In the loop region, from A65 to S85, structure **302a** showed the highest RMSF value (approximately 0.9 nm) compared with structures **135** and **302a**, which reached RMSF values lower than 0.6 nm. This behavior might be due to differences in the spatial conformation of **302a** within the active site of *Lb*PTR1 compared with those for structures **135** and **302**; consequently, the molecular docking values are justified. The analysis of the loop section between N110 and T135 showed that inhibitors (structures **135**, **302**, and **302a**, DHB, and PMA) in complex with *Lb*PTR1 reached RMSF values approaching 1.0 nm; in contrast, the apoenzyme exhibited a value above 1.65 nm (Figure 6b), indicating that these structures stabilized the protein following the formation of an *Lb*PTR1–kaurane complex. The diterpenes showed similar RMSF values as DHB; however, in this loop region, PMA has a lower RMSF value (approximately 0.8 nm).

The evolution of the *Lb*PTR1 packing level was observed through the radius of gyration (RoG) values. The diterpene–*Lb*PTR1 complexes showed no differences in RoG values compared with the complex formed between DHB and *Lb*PTR1 (RoG of approximately 2.00 nm), with fluctuations in the tertiary structure lower than 0.10 nm. The RoG values for PMA were slightly different (approximately 2.05), demonstrating a different behavior throughout the 50 ns test period than the other structures analyzed (Figure 6c). Apo*Lb*PTR1, during the initial 25 ns, showed a higher RoG than the complexes, with a RoG value approaching 2.05 nm. However, in the second half of the simulation, a decrease in the RoG value was observed, reaching a value similar to those observed for the complexes formed with diterpenes and DHB (RoG of approximately 1.95 nm). Based on these results, the structures **135**, **302**, and **302a** appeared to be stably folded after the MD simulation.

According to the MD simulations, the binding free energies for structures **135**, **302**, **302a**, PMA, and DHB were calculated through the MM/PBSA method. The diterpenes **135**, **302**, and **302a** presented binding free energy values of −132.7 kJ/mol, −121.4 kJ/mol, and −138.3 kJ/mol, respectively, which were all lower energy values than those measured for DHB and PMA in complex with *Lb*PTR1, which presented free energy values of −107.4 kJ/mol and −110.0 kJ/mol, respectively. These differences in energetic contributions were associated with structural differences (Table 6).

In the three kauranes, van der Waals interactions showed the highest negative contributions to the binding free energy, which supported the previously observed docking results. The solvent-accessible surface area (SASA) and electrostatic parameters contributed negatively, but to a lesser degree, to the binding free energies in similar proportions (except for the electrostatic parameter of **302**, which displayed a higher contribution to total binding energy). The reference inhibitor (PMA) and the native ligand (DHB) demonstrated different behaviors from those observed for the three diterpenes, with a higher contribution of electrostatic interactions to the total binding free energy value, which represented the energy parameter with the highest negative energetic contribution. For all molecules, the polar solvation had a positive contribution to the total energy value, with larger contributions to the complexes DHB-*Lb*PTR1 and PMA-*Lb*PTR1.

### 2.5. Prediction of ADMET Properties

The pharmacokinetic properties such as absorption, distribution, metabolism, excretion, and toxicity (ADMET) of the structures **135**, **302,** and **302a** were predicted by using Volsurf+ [31,57], ADMETlab 2.0 [58], and OSIRIS Data Warrior v.5.2.1 [59]. The results show that all three structures did not present any violation to the Lipinski’s “rule of five”, implying that these kauranes are probably orally bioavailable. These compounds also showed a high gastrointestinal absorption (Table S6).

Cytochrome P450 (CYP) enzymes are a family of heme proteins involved in the metabolism of frequent pharmacologically active compounds and can cause drug to drug interactions with co-administered drugs as well as unwanted adverse side effects [60]. The results obtained in ADMETlab 2.0 [58] showed that structures **135**, **302,** and **302a** are substrates of CYP2C19. Additionally, structures **302** and **302a** presented probabilities above 0.53 of being substrate of CYP2C9 and the structure **135** is substrate of CYP2D6 and CYP3A2. All selected kauranes are non-inhibitors of the studied CYP 450 isoenzymes.

That being said, these molecules do not affect significantly the metabolism of the drugs that work as substrates of the mentioned isoenzymes, therefore being safe to use concomitantly with additional pharmacotherapy (Table S6).

Structures **135** and **302a** had no predicted risk for the development of mutagenicity, tumorigenesis, negative effects on the reproductive system, or irritability; only structure 302 was predicted as potentially irritable. The identification of potential hERG channel blockers at the early stage of drug discovery process will decrease the risk of cardiotoxicity-related attritions in the later and more expensive development stage [61]. The three evaluated kauranes (structures **135**, **302,** and **302a**) showed minimal probabilities of being hERG blockers with values lower than 0.022 (Table S6). Finally, using the webtools CLC-Pred [62] and eMolTox [63], cytotoxic properties were predicted against no tumor cell lines; for the three structures from data-driven models no toxic action was found.

## 3. Materials and Methods

### 3.1. Database

From the ChEMBL database (https://www.ebi.ac.uk/chembl/, accessed on 10 January 2021), we selected a diverse set of 1085 structures that were initially classified according to their predicted activity against *L. major*. These compounds were classified according to their pIC_50_ values [−logIC_50_ (mol/L)]; therefore, we stratified them into active (pIC_50_ ≥ 5.0) and inactive (pIC_50_ < 5.0) structures.

The APD, based on Euclidean distances, was used to identify those compounds in the test set for which predictions may be unreliable; compounds were considered unreliable if they had APD values higher than d + Zσ, where d is the average Euclidian distance, and σ is the standard deviation of the set of samples used as the training set, with lower-than-average Euclidian distance values relative to all samples in the training set. The parameter Z is an empirical cutoff value, and 0.5 was used as the default value [64].

Structures with pIC_50_ values ranging from 4.9 to 5.0 (range of 0.1 units) were excluded to avoid edge effects and improve the predictive capacity of the models. Excluding these structures minimized the differences in activity values resulting from errors and differences in experimental protocols [65]. Data curation was performed for the datasets according to procedures suggested in the literature [66,67,68]. Standardizer software [JChem, version 16.11.28 (2016), calculation module developed by ChemAxon, https://www.chemaxon.com/, accessed on 20 January 2021] was used to canonize all simplified molecular-input line-entry system (SMILES) codes. After duplicate structures were removed, those with higher pIC_50_ values were eliminated, facilitating the generation of more restrictive models. Finally, 638 structures for *L. major* (338 active and 300 inactive structures) were included in the analysis.

The kaurane dataset was built in-house, and a total of 360 molecules from this dataset were used in this study. For all structures, SMILES codes were used as the input data in Marvin [ChemAxon, version 16.11.28 (2016), calculation module developed by ChemAxon, https://www.chemaxon.com/, accessed on 20 January 2021]. We used standardizer software [JChem, version 16.11.28 (2016), calculation module developed by ChemAxon, https://www.chemaxon.com/, accessed on 20 January 2021]. ChemAxon was used to canonize the structures, add hydrogens, perform aromatic form conversions, and clean molecular graphs in three dimensions.

### 3.2. Volsurf+ Descriptors

The three-dimensional (3D) structures of the identified molecules, in special data file (SDF) format, were used as the input data for VolSurf+, v. 1.0.7 [30] and were subjected to molecular interaction fields (MIFs) to generate descriptors using the following probes: N1 (amide nitrogen–hydrogen bond donor probe), O (carbonyl oxygen–hydrogen bond acceptor probe), OH2 (water probe), and DRY (hydrophobic probe). Additional non-MIF-derived descriptors were generated, resulting in a total of 128 descriptors [30]. One of the main advantages of using VolSurf descriptors is the relatively low influence of conformational sampling and averaging on these descriptors [31].

### 3.3. RF Models

Knime 3.1.0 software (KNIME 3.1.0 the Konstanz Information Miner Copyright, 2003–2014, www.knime.org) [32] was used to perform all of the following analyses. Initially, the descriptors calculated in the VolSurf+ program were imported in comma-separated value (CSV) format, and the “Partitioning” node in the stratified sampling option was used to classify 80% of the initial dataset as the training set and the remaining 20% as the test set. The model was generated by employing the modeling set and the RF algorithm, with a five-fold cross-validation procedure, using WEKA nodes. In the five-fold cross-validation procedure, the dataset is divided five times into a modeling set (80%/20%). The modeling set (which was used to build and validate the models) was further divided into training (80%) and test sets (20%) [33,66]. The parameters selected for the RF models included the following: number of trees to build = 200; seed for random number generator = 1; and Gini Index, as a split criterion, for both the training and internal cross-validation sets. From the confusion matrix, the internal and external performances of the selected models were analyzed, using the following parameters: sensitivity (true-positive rate), specificity (true-negative rate), and accuracy (overall predictability). In addition, to describe the true performance of the model with more clarity than can be obtained from accuracy alone, the ROC curve was employed, using a “ROC curve” node, which uses the sensitivity and specificity parameters. The plotted ROC curve shows the true-positive (active) rate versus the false-positive rate (1 − specificity) [34]. In this representation, when a variable of interest cannot be distinguished between the two groups, the AUC value is 0.5, whereas a perfect separation between the values of the two groups, with no distribution overlap, results in an AUC value of 1. The MCC was also calculated, for which a value of 1 represents a perfect prediction, a value of 0 represents a random prediction, and a value of −1 represents a total disagreement between the prediction and the observation [35].

### 3.4. Synthesis of VS-Selected Diterpenes

#### 3.4.1. Materials and Reagents

Optical rotations and UV data were recorded using a Jasco P-2000ST digital polarimeter (Jasco, Tokyo, Japan) and a Thermo Fisher Scientific Genesys 10S spectrophotometer (Thermo Fisher Scientific, Waltham, MA, USA), respectively. ^1^H and ^13^C Nuclear magnetic resonance experiments were recorded in a Bruker Avance400 spectrometer (Bruker Corporation, Billerica, MA, USA) using CDCl_3_ as solvent. All shifts are given in δ (ppm) using the signal of TMS as reference. All coupling constants (*J*) are given in Hz. HRESIMS data were obtained on a Bruker micro-QToF II spectrometer (Bruker, Billerica, MA, USA), respectively. Thin-layer chromatography (TLC) using silica gel 60 F254 TLC plates (Merck, Darmstadt, Germany) and mobile phases comprising solvent mixtures of n-hexane, EtOAc, and MeOH were used. Plates after TLC development were observed under UV light (254 and 365 nm) and derivatized using I_2_ vapor and Hannessian’s reagent (aqueous solution of ammonium molybdate, cerium sulphate and H_2_SO_4_). Silica gel (SiO2) 60 (0.04–0.063 mm) (Merck) was used for flash chromatography (flash CC). Cinnamic acid, *p*-coumaric acid, (R)-(−)-carvone, and other reagents and solvents for synthesis and enzyme inhibition assay were acquired from Sigma-Aldrich (St. Louis, MO, USA).

#### 3.4.2. Isolation of Compounds **A**–**C**

Fruits (325 g) of Xylopia frutescens (Annonaceae) were extracted with ethanol 96% and a portion of the resulting crude extract (25.5 g) was fractionated by CC over SiO_2_ in gradient elution (n-hexane to methanol) affording twenty-five different fractions. Fractions 7, 8, 11, and 13 were independently depurated by flash CC on SiO_2_, yielding compounds *ent*-kaurenoic acid (**A**) (75.6 mg) [42], 3α-hydroxy-*ent*-kaur-16-en-19-oic acid (**B**) (52.3 mg) [41], and 3α,9β-dihydroxy-*ent*-kaur-16-en-19-oic acid (**C**) (42.6 mg), using mixtures of *n*-hexane:EtOAc:MeOH 9:1.5:0.5; 8:1:1, 7:2:1, and 6:2:2, respectively.

3α,9β-dihydroxy-*ent*-kaur-16-en-19-oic acid (**C**): Oil; $[\alpha {]}_{D}^{20}$ = −61.8 (c = 0.1, CHCl_3_); ^1^H NMR (400 MHz, CDCI_3_) δ 4.78 (br s, 1H, H-17), 4.67 (br s, 1H, H-17), 4.63 (dd, *J* = 12.0, 5.0 Hz, 1H, H-3), 2.65 (br s, 1H, H-13), 2.63 (br d, *J* = 13.5 Hz, 1H, H-15b), 2.49–2.44 (m, 1H, H-2a), 2.31 (dd, *J* = 11.0, 1.3 Hz, 1H, H-14b), 2.16–2.10 (m, 2H, H-1a, H-7a), 2.07–2.03 (m, 1H, H-12b), 1.88–1.85 (m, 1H, H-5), 1.82–1.76 (m, 2H, H-6a, H-15a), 1.73–1.67 (m, 1H, H-2b), 1.65–1.55 (m, 4H, H-11b, H-14a, H-6b, H-1b), 1.48–1.44 (m, 2H, H-11a, H-12a), 1.35–1.33 (m, 1H, H-7b), 1.29 (s, 3H, H-18), 1.15 (s, 3H, H-20); ^13^C NMR (100 MHz, CDCI_3_) δ 180.1 (C-19), 156.8 (C-16), 104.4 (C-17), 78.6 (C-3), 76.9 (C-9), 50.3 (C-8), 49.4 (C-5), 48.5 (C-4), 48.5 (C-15), 43.6 (C-10), 42.1 (C-13), 39.6 (C-14), 35.2 (C-7), 34.5 (C-1), 30.5 (C-12), 29.6 (C-11), 24.3 (C-18), 24.2 (C-2), 20.9 (C-6), 17.4 (C-20); HRESIMS [M + H]^+^ *m/z* 335.2203 (calcd. for C_20_H_31_O_4_, 335.2222).

#### 3.4.3. Synthesis of 2β-Hydrohy-menth-6(1)-en-5β-yl ent-kaurenoate (**135**)

The synthesis of the top-ranked ester 135 was accomplished following the next four synthetic steps:

##### Synthesis of 5β-Hydroxy-(*R*)-carvone (**E**)

Compound G was synthesized as previously reported [44]. Briefly, Cu–Al Ox catalyst (168 mg) was placed into a 100-mL round-bottom flask (RBF) containing absolute EtOH (30 mL). The resulting mixture was stirred at room temperature (rt) for 10 min. Subsequently, (*R*)-(−)-carvone (D) (450 mg, 3.0 mmol, 1.0 equiv) and *t-*BuOK (168 mg, 1.5 mmol, 0.5 equiv) were added, and this reaction mixture was further stirred for 30 h at rt. After completion, the mixture was filtered through celite, rinsing with MeOH (15 mL). The filtrate was concentrated under reduced pressure, and the residue was purified by flash CC on SiO_2_ (10% EtOAc in *n*-hexane) to afford compound E (194 mg, 39% yield). Oil; $[\alpha {]}_{D}^{20}$ +65.2 (c 0.1, CHCI_3_); ^1^H NMR (400 MHz, CDCl_3_) δ 6.85 (br d, *J* = 9.7 Hz, 1H), 5.05 (br s, 2H), 4.48 (dd, *J* = 9.7, 1.9 Hz, 1H), 3.29 (ddd, *J* = 6.5, 2.6, 1.9 Hz, 1H), 2.65 (dd, *J* = 11.8, 2.6 Hz, 1H), 2.29 (dd, *J* = 11.8, 6.5 Hz, 1H), 1.79 (s, 3H), 1.77 (s, 3H); ^13^C NMR (100 MHz, CDCl_3_) 198.5, 147.7, 437, 135.3, 114.6, 68.4, 52.7, 40.5, 19.1, 15.3; HRESIMS [M + H]^+^ *m/z* 167.1055 (calcd for C_10_H_15_O_2_, 167.1072).

##### Synthesis of 2-Oxo-menth-6-en-5β-ol (**F**)

Compound **F** was prepared as previously reported [44]. Briefly, RhCl (PPh_3_)_3_ (46.2 mg, 0.05 mmol, 5 mol%) was added to a 25-mL RBF containing a stirred solution of E (166 mg, 1.0 mmol, 1.0 equiv.) in dry toluene (10 mL) under nitrogen. This flask was sealed with a rubber septum, headspace evacuated, and hydrogen flushed. The reaction mixture was stirred at rt for 14 h. After completion, the solvent was removed under reduced pressure and the residue was purified by flash CC on SiO_2_ (5% EtOAc in *n*-hexane) to afford compound **F** (153 mg, 91% yield). Oil; $[\alpha {]}_{D}^{20}$ = −62.1 (c = 0.3, CHCl_3_); ^1^H NMR (400 MHz, CDCI_3_) δ 6.83 (dd, *J* = 9.0, 1.3 Hz, 1H), 4.31 (d, *J* = 9.5 Hz, 1H), 2.48 (dd, *J* = 15.5, 3.6 Hz, 1H), 2.13–2.09 (m, 3H), 2.01–1.87 (m, 1H), 1.75 (s, 3H), 0.95 (d, *J* = 7.1 Hz, 3H), 0.88 (d, *J* = 7.0 Hz, 3H); ^13^C NMR (100 MHz, CDCI_3_) δ 200.5, 148.8, 135.1, 69.5, 50.8, 37.2, 26.6, 20.5, 16.6, 15.5; HRESIMS [M + H]^+^ *m/z* 169.1211 (calcd. for C_10_H_17_O_2_, 169.1229).

##### Synthesis of 2-Oxo-menth-6-en-5β-yl ent-kaurenoate (**G**)

Compound **G** was obtained by Steglich esterification [45] from **A** and **F**. Briefly, *ent*-kaurenoic acid (**A**) (30.2 mg, 0.1 mmol, 1.0 eq), compound **F** (16.6 mg, 0.1 mmol, 1.0 eq), and dimethylaminopyridine (DMAP) (2.5 mg, 0.02 mmol, 0.2 eq) were mixed within a 10-mL RBF. This flask was sealed with a rubber septum, inner air evacuated, and nitrogen flushed. Anhydrous CH_2_Cl_2_ (3 mL) was added, followed by 1 M dicyclohexylcarbodiimide (DCC) in CH_2_Cl_2_ (110 µL, 0.11 mmol, 1.10 eq). The resulting mixture was stirred overnight then filtered through celite. The filtrate was concentrated under reduced pressure and the residue was purified by flash CC on SiO_2_ (20% EtOAc in *n*-hexane) to afford compound **G** (35.7 mg, 79% yield). Oil; $[\alpha {]}_{D}^{20}$ = −58.5 (c = 0.2, CHCl_3_); ^1^H NMR (400 MHz, CDCl3) δ 6.79 (br d, *J* = 9.4 Hz, 1H), 5.21 (dd, *J* = 9.4, 1.7 Hz, 1H), 4.78 (br s, 1H), 4.72 (br s, 1H), 2.76–2.68 (m, 2H), 2.65–2.60 (m, 2H), 2.17 (br d, *J* = 13.0 Hz, 1H), 2.08–2.03 (m, 2H), 1.88 (dd, *J* = 11.4, 1.1 Hz, 1H), 1.16 (dd, *J* = 11.4, 4.7 Hz, 1H), 1.91–1.83 (m, 5H), 1.78 (s, 3H), 1.47–1.40 (m, 1H), 1.59–1.51 (m, 6H), 1.16 (s, 3H), 1.08–1.05 (m, 1H), 1.05–1.03 (m, 1H), 1.02 (d, *J* = 6.7 Hz, 3H), 1.01–0.97 (m, 1H), 0.97 (d, *J* = 6.7 Hz, 3H), 0.88 (s, 3H), 0.76 (m, 1H); 13C NMR (100 MHz, CDCI_3_) δ 200.6, 177.9, 155.5, 140.1, 134.4, 102.4, 71.5, 57.8, 55.2, 49.5, 45.6, 44.7, 44.4, 43.5, 41.4, 40.2, 39.4, 38.9, 37.7, 37.3, 33.5, 28.6, 26.8, 21.5, 20.3, 19.1, 18.8, 16.7, 15.9, 15.7; HRESIMS [M + H]^+^ *m/z* 453.3345 (calcd for C_30_H_45_O_3_, 453.3369).

##### Synthesis of 2β-Hydroxy-menth-6-en-5β-yl ent-kaurenoate (**135**)

Compound **135** was obtained from G, through Luche reduction using a reported procedure [69]. Briefly, compound **G** (27.1 mg, 0.06 mmol, 1.0 eq), CeCl_3_.7H_2_O (5.6 mg, 0.015 mmol, 0.25 eq), and MeOH (3 mL) were mixed into a 10-mL RBF by stirring at 0 °C. A 1 M NaBH_4_ solution (0.06 mL, 0.06 mmol) in MeOH was then added. Reaction mixture was allowed to warm to 20 °C and then stirred at this temperature for 1 h. After completion, reaction was quenched with 2M HCl (2 mL) and extracted with CH_2_Cl_2_ (3 × 2 mL). The separated CH_2_Cl_2_ extract was washed with 10% NaCl (2 × 3 mL), dried over MgSO_4_, filtered, and concentrated under reduced pressure. The resulting residue was purified by flash CC on SiO_2_ (20% EtOAc in *n*-hexane) to afford 135 (22.4 mg, 82% yield) (wedelobatin A) [70]. Oil; $[\alpha {]}_{D}^{20}$ −92.6 (c 0.2, CHCI_3_); ^1^H NMR (400 MHz, CDCl_3_) δ 5.44 (br s, 1H), 5.17 (br d, *J* = 8.5 Hz, 1H), 4.77 (br s, 1H), 4.75 (br s, 1H), 4.03 (t, *J* = 3.2 Hz, 1H), 2.63 (br s, 1H), 2.19 (br d, *J* = 13.3 Hz, 1H), 2.06–2.02 (m, 2H), 1.93 (dd, *J* = 11.1, 1.2 Hz, 1H), 1.14 (dd, *J* = 11.1, 5.0 Hz, 1H), 1.87–1.81 (m, 7H), 1.79 (s, 3H), 1.46–1.41 (m, 2H), 1.60–1.50 (m, 6H), 1.19 (s, 3H), 1.07–1.05 (m, 1H), 1.03–1.01 (m, 1H), 1.00–0.97 (m, 1H), 0.95 (d, *J* = 6.8 Hz, 3H), 0.90 (s, 3H), 0.81 (d, *J* = 6.8 Hz, 3H), 0.78 (m, 1H); ^13^C NMR (100 MHz, CDCI_3_) δ 177.5, 155.4, 139.4, 124.4, 101.9, 71.6, 67.4, 57.2, 55.6, 48.8, 44.2, 43.9, 43.4, 41.1, 41.2, 40.1, 40.1, 39.4, 37.7, 33.0, 30.2, 29.2, 26.2, 22.2, 21.1, 20.2, 19.2, 18.2, 17.2, 16.0; HRESIMS [M + H]^+^ *m/z* 455.3511 (calcd for C_30_H_47_O_3_, 455.3525).

##### 3.4.4. Synthesis of 3α-Cinnamoyloxy-9β-hydroxy-ent-kaur-16-en-19-oic Acid (**301**), 3α-cinnamoyloxy-ent-kaur-16-en-19-oic Acid (**302**), 3α-p-coumaroyloxy-9β-hydroxy-ent-kaur-16-en-19-oic Acid (**301a**), 3α-p-coumaroyloxy-ent-kaur-16-en-19-oic Acid (**302a**)

Separated reaction, following the same procedure as described for compound **H** (Steglich esterification [45]) of cinnamic acid (H) (7.4 mg, 0.05 mmol, 1.0 eq) with B (15.9 mg, 0.05 mmol, 1.0 eq) or C (16.7 mg, 0.05 mmol, 1.0 eq) afforded the top-ranked compounds 301 (17.9 mg, 77%) and 302 (17.5 mg, 78%), respectively. Additionally, separated reaction of *p*-coumaric acid (I) (8.2 mg, 0.05 mmol, 1.0 eq) with B (15.9 mg, 0.5 mmol, 1.0 eq) and C (16.7 mg, 0.5 mmol, 1.0 eq) afforded compounds 301a (17.0 mg, 71%) and 302a (16.0 mg, 69%), respectively.

**301**: Oil; $[\alpha {]}_{D}^{20}$ −56.3 (c 0.05, CHCI_3_); ^1^H NMR (400 MHz, CDCl_3_) δ 7.68–7.66 (m, 2H), 7.65 (d, *J* = 15.3 Hz, 1H), 7.40–7.37 (m, 3H), 6.49 (d, *J* = 15.3 Hz, 1H), 4.82 (br s, 1H), 4.77 (br s, 1H), 4.68 (dd, *J* = 12.2, 4.5 Hz, 1H), 2.71 (br d, *J* = 14.7 Hz, 1H), 2.59 (br s, 1H), 2.53–2.50 (m, 1H), 2.28 (dd, *J* = 10.5, 1.8 Hz, 1H), 2.16–2.09 (m, 2H), 2.04–2.00 (m, 1H), 1.93–1.80 (m, 3H), 1.74–1.70 (m, 1H), 1.67–1.55 (m, 3H), 1.51 (dd, *J* = 10.5, 5.3 Hz, 1H), 1.48–1.43 (m, 2H), 1.30–1.27 (m, 1H), 1.26 (s, 3H), 1.12 (s, 3H); ^13^C NMR (100 MHz, CDCI_3_) δ 179.7, 166.7, 157.9, 145.1, 134.7, 130.1, 128.7, 128.2, 118.6, 105.8, 78.5, 75.4, 52.3, 52.1, 49.8, 49.3, 43.7, 42.3, 38.9, 38.5, 34.6, 30.7, 27.3, 25.5, 24.3, 20.5, 17.8; HRESIMS [M + H]^+^ *m/z* 465.2623 (calcd for C_29_H_37_O_5_, 465.2641).

**302**: Oil; $[\alpha {]}_{D}^{20}$ −41.2 (c 0.03, CHCI_3_); ^1^H NMR (400 MHz, CDCl_3_) δ 7.69 (d, *J* = 15.1 Hz, 1H), 7.65–7.62 (m, 2H), 7.47–7.43 (m, 3H), 6.54 (d, *J* = 15.1 Hz, 1H), 4.81 (br s, 1H), 4.75 (br s, 1H), 4.64 (dd, *J* = 12.1, 4.7 Hz, 1H), 2.66 (br s, 1H), 2.36–2.32 (m, 1H), 2.07–2.03 (m, 2H), 1.96 (d, *J* = 11.1 Hz, 1H), 1.93–1.90 (m, 1H), 1.84–1.81 (m, 1H), 1.68–1.62 (m, 3H), 1.55–1.50 (m, 3H), 1.47–1.42 (m, 2H), 1.13–1.07 (m, 2H), 1.05 (br s, 1H), 1.01 (d, *J* = 9.3 Hz, 1H), 1.21 (s, 3H), 0.97 (s, 3H); ^13^C NMR (100 MHz, CDCI_3_) δ 180.2, 166.8, 155.1, 145.3, 134.6, 130.3, 128.8, 128.1, 118.5, 103.1, 79.1, 75.1, 56.5, 48.6, 48.3, 43.8, 43.5, 41.3, 39.6, 39.5, 38.8, 33.3, 24.3, 23.7, 21.5, 18.5, 15.5; HRESIMS [M + H]^+^ *m/z* 449.2678 (calcd for C_29_H_37_O_4_, 449.2692).

**301a**: Oil; $[\alpha {]}_{D}^{20}$ −43.7 (c 0.03, CHCI_3_); ^1^H NMR (400 MHz, CDCl_3_) δ 7.71 (d, *J* = 15.0 Hz, 1H), 7.48 (d, *J* = 8.3 Hz, 2H), 6.91 (d, *J* = 8.3 Hz, 2H), 6.38 (d, *J* = 15.0 Hz, 1H), 4.84 (br s, 1H), 4.71 (br s, 1H), 4.66 (dd, *J* = 11.9, 4.9 Hz, 1H), 2.69 (d, *J* = 14.5 Hz, 1H), 2.57 (br s, 1H), 2.55–2.51 (m, 1H), 2.26 (dd, *J* = 10.8, 1.6 Hz, 1H), 2.20–2.17 (m, 1H), 2.15–2.11 (m, 1H), 2.03–1.98 (m, 1H), 1.91–1.80 (m, 3H), 1.78–1.76 (m, 1H), 1.69–1.63 (m, 2H), 1.58–1.55 (m, 1H), 1.53 (dd, *J* = 10.6, 5.1 Hz, 1H), 1.49–1.44 (m, 2H), 1.29–1.26 (m, 1H), 1.24 (s, 3H), 1.13 (s, 3H); ^13^C NMR (100 MHz, CDCI_3_) δ 181.1, 166.1, 158.1, 156.6, 144.2, 129.1, 127.1, 118.3, 117.9, 107.1, 78.5, 75.4, 52.3, 52.1, 49.6, 49.5, 43.4, 41.8, 38.7, 38.4, 34.5, 30.7, 27.6, 25.7, 24.3, 20.4, 17.9; HRESIMS [M + H]^+^ *m/z* 481.2577 (calcd for C_29_H_37_O_6_, 481.2590).

**302a**: Oil; $[\alpha {]}_{D}^{20}$ −36.5 (c 0.01, CHCI_3_); ^1^H NMR (400 MHz, CDCl_3_) δ 7.76 (d, *J* = 15.2 Hz, 1H), 7.51 (d, *J* = 8.1 Hz, 2H), 6.87 (d, *J* = 8.1 Hz, 2H), 6.45 (d, *J* = 15.2 Hz, 1H), 4.85 (br s, 1H), 4.77 (br s, 1H), 4.62 (dd, *J* = 12.0, 4.9 Hz, 1H), 2.67 (br s, 1H), 2.38–2.34 (m, 1H), 2.10–2.06 (m, 2H), 1.97 (d, *J* = 11.4 Hz, 1H), 1.94–1.90 (m, 1H), 1.85–1.81 (m, 1H), 1.69–1.65 (m, 2H), 1.61–1.53 (m, 4H), 1.49–1.44 (m, 2H), 1.17–1.13 (m, 1H), 1.09–1.07 (m, 1H), 1.04 (br s, 1H), 1.02 (d, J = 9.5 Hz, 1H), 1.21 (s, 3H), 0.97 (s, 3H); ^13^C NMR (100 MHz, CDCl_3_) δ 180.5, 166.3, 158.3, 155.7, 144.8, 129.5, 127.7, 118.5, 117.4, 103.1, 79.1, 75.5, 56.7, 48.7, 48.1, 44.2, 43.8, 41.1, 40.3, 39.3, 39.3, 33.2, 24.5, 23.4, 21.5, 18.7, 15.4; HRESIMS [M + H]^+^ *m/z* 465.2628 (calcd for C_29_H_37_O_5_, 465.2641).

### 3.5. LmPTR1 Enzyme Inhibition Assay

Recombinant *Lm*PTR1 enzyme was obtained, purified, and kinetically characterized, as reported previously [71]. The in vitro assessment of selected diterpenes (i.e., **135**, **301**, **302**, **301a**, and **302a**) for *Lm*PTR1 inhibitory activity was performed through the spectrophotometric monitoring of the enzymatic activity under balanced conditions: *Lm*PTR1 (30 µg), 7,8-dihydro-L-biopterin (DHB, 20 µM), sodium citrate buffer (20 mM, pH 6.0), 30 °C, and a final assay volume of 600 µL. Each reaction was started by the addition of 250 µM NADPH. Absorbance was monitored at 340 nm (i.e., oxidation of NADPH to NADP+) for 240 s, and the resulting profile was used to measure the initial reaction rate (IRR) through the respective slope by linear regression. All recordings were performed in triplicate. PMA was used as the positive control. The resulting IRR values were used to calculate the % inhibition, as 100 − (Ri/Rc × 100), where Ri is the IRR in the presence of the inhibitor and Rc is the IRR in the absence of inhibitors (1% DMSO *v/v* final concentration). The % inhibition for at least five concentrations (range: 0.1–128 µM) for each test compound (diterpenes and PMA) were calculated, and concentration-response curves (% inhibition vs. Log[inhibitor]) were obtained by non-linear regression to determine the IC_50_ using GraphPad Prism 5.0 (GraphPad, San Diego, CA, USA). Finally, Ki^app^ values were calculated using the Cheng–Prusoff equation for competitive inhibition, assuming a 1:1 stoichiometry and that the inhibitor-binding reactions are reversible [47]: Ki^app^ = IC_50_/(1 + [S]/*Km*), where [S] is the substrate (DHB) concentration and *Km* is the Michaelis constant. The substrate *Km* was calculated during the kinetic characterization of purified, recombinant *Lm*PTR1.

### 3.6. Hybrid Models of L. braziliensis, L. panamensis and L. amazonensis

Hybrid models for *Lb*, *Lp,* and *La*PTR1 were constructed using YASARA software (YASARA (18.4.24) Vienna, Austria: YASARA Biosciences GmbH; 2018) based on the FASTA sequences of *Lb*PTR1 (A4HCP1), *Lp*PTR1 (A0A088SA10), and *La*PTR1 (O09352), which were obtained from the UniProt database (https://www.uniprot.org/, accessed on 9 February 2021). The stereochemical qualities of the models were evaluated with PROCHECK [72], in which molecular diversity evaluated several stereochemical parameters, such as the torsional angles of the main chain, the torsional angles of the side chain, bad contacts or steric impediments, and planarity. PROCHECK generated a Ramachandran graph [51], which verified the allowed and unallowed regions of the main amino acid chain. The structural quality was evaluated in VERIFY 3D software (https://services.mbi.ucla.edu/SAVES/, accessed on 13 February 2021), which analyzes the compatibility of the protein sequence with its 3D structure, according to the chemical environment, and WHAT IF (https://swift.cmbi.ru.nl/servers/html/index.html, accessed on 15 February 2021), which analyzes various structural parameters, such as the atomic contacts between residues. The software Discovery Studio Visualizer was used to visualize the modeled protein [73].

### 3.7. Molecular Docking Calculations

The *Lm*PTR1 crystal structure (PDB ID: 1E7W), in complex with its respective inhibitor, methotrexate (PDB ID: MTX), was downloaded from PDB [38]. Using Molegro 6.0.1 software, all water compounds were deleted from the enzyme structures, and the enzyme/compound structures were prepared using the same default parameter settings, in the same software package (Score function: MolDock Score; Ligand evaluation: Internal ES, Internal H-Bond, Sp2–Sp2 Torsions, all checked; Number of runs: 10 runs; Algorithm: MolDock SE; Maximum Interactions: 1500; Max. population size: 50; Max. steps: 300; Neighbor distance factor: 1.00; Max. number of poses returned: 5). The docking procedure was performed using a grid with a 15-Å radius and a 0.30-Å resolution to cover the ligand-binding site for the four enzyme structures [14,20].

The docking procedures for hybrid models of *Leishmania* (*Lb*, *Lp,* and *La*) were performed with the Autodock/Vina (1.1.2) plug-in for PyMOL (1.3r2), under a Python 2.5.2 environment for Windows. Docking calculations were then performed between the minimized ligand through a cube (dimensions 22.5 Å × 22.5 Å × 22.5 Å, grid spacing 0.375 Å) located in the geometric center of the binding pocket (coordinates *Lb*: 18.75, −13.1, 10.25; *Lp*: 18.1, 12.6, 8.0; and *La*: 20.1, 19.6, 7.8), which was identified through cavities analysis in Molegro 6.0.1. Flexible residues in the binding site were selected for each model. *Lb*: L19, H39, R40, N110, S112, D181, and S227; *Lp*: K17, L19, S112, M179, and I180; and *La*: R18, L19, H38, L188, M233, K244, and Y283. Docking poses were classified according to their docking scores (such as the free energy or affinity). Each calculation was performed in three replicates. Two known PTR1 ligands (DHB and PMA) were used as controls. The two-dimensional (2D)-residual interaction diagrams were visualized on Discovery Studio 2016 Visualizer Client (Biovia, San Diego, CA, USA) [73].

### 3.8. Molecular Dynamics Simulations

MD simulations were run in the Gromacs 5.0.5 on Ubuntu 12.04 server [74,75]. Structures **135**, **302**, and **302a** displayed the best poses from docking, and the DHB and PMA structures, as well as the hybrid model of *Lb*PTR1, were employed as the inputs for the MD simulations. The five ligands were prepared by adding hydrogen atoms and the corresponding charges using the AM1-BCC charge scheme in UCSF Chimera. Subsequently, ligand topologies were generated automatically with ACPYPE script. Protein topologies were obtained in Gromacs using the Amber 99SB force field, and the TIP3P water model was implemented. Solvation was performed in a triclinic box using a margin distance of 1.0 nm. The addition of 0.1 M NaCl to complexes and proteins was performed by randomly replacing water molecules until neutrality was achieved [20,56].

The systems were energy-minimized by 2000 steps using the steepest descent method. Systems were subjected to NVT equilibration performed at 310 K for 50 ps, followed by NPT equilibration for 500 ps, using the Parrinello−Rahman method at 1 bar as a reference, using position restraints. Finally, the solute position restraints were released, and a production run for 5 ns was performed. The temperature and pressure were maintained constant at 310 K and 1 bar, respectively. Coordinates were recorded in a 1 fs time step. Electrostatic forces were calculated using the particle-mesh Ewald method. Periodic boundary conditions were used in all simulations, and covalent bond lengths were constrained by the LINCS algorithm. The molecular mechanics Poisson–Boltzmann surface area (MM/PBSA) method was used to calculate binding free energies, using the trajectories calculated by the MD simulations [20,56].

### 3.9. Prediction of ADMET Properties

For the structures **135**, **301**, and **302a**, the ADMET parameters were calculated using the ADMETlab 2.0, an integrated online platform for predictions of ADMET properties [58]. Drug toxicity prediction was performed using OSIRIS Data Warrior v.5.2.1, based on the following parameters: mutagenicity, tumorigenicity, reproductive effect, and irritability [59]. For in silico prediction of human cell line cytotoxicity, two web-tools were used: CLC-Pred, a freely available web-service [62] and eMolTox, a web server for the prediction of potential toxicity associated with a given molecule [63].

## 4. Conclusions

Structures **135** and **302** are two kauranes that were identified as hits for anti-leishmanicidal activity, with IC_50_ values against *L. major* below 10 μM. These two structures were selected from an in-house database comprising 360 kauranes through an in silico approach combining machine learning and molecular docking methodologies. Only five structures from Asteraceae were classified as active by both methodologies. The in vitro results allowed the successful verification of the RF classification model, which predicted that structures **135** and **302** would be active (pIC_50_ > 5.0) and that structure **301** would be inactive (pIC_50_ < 5.0), which was observed experimentally.

Additionally, the inhibitory activity was improved by approximately 60% when a 3α-*p*-coumaroyloxy group was used in **302** in place of the 3α-cinnamoyloxy substituent, with **302a** exhibiting a lower Ki^app^ value. Although the tested diterpenes were found to be less active than the positive control, the validity of the designed VS approach for the selection of bioactive molecules against PTR1 was demonstrated, and the computationally studied binding mode of these selected compounds within the active site of *Lm*PTR1, which causes CL, was explored. These selected compounds can be considered important leads that can be used to obtain more active PTR1 inhibitors.

Finally, because throughout the American continent, other *Leishmania* species are responsible for the clinical diversity of CL and MCL, including *L. amazonensis* (*La*), *L. braziliensis* (*Lb*), and *L. panamensis* (*Lp*), molecular docking calculations and MD simulations were performed for the entire set of kauranes (including **301a** and **302a**), and the compounds **135**, **302**, and **302a** were identified as potential multispecies agents. Therefore, this study describes a valuable screening approach for the identification of lead compounds in natural products, which can contribute to the further development of alternative chemotherapies against this group of diseases.

## Figures and Tables

**Table 1:** Docking energies of the best-ranked structures from the structure-based VS for *L. major* PTR1. SD = standard deviation; RMSD values = root mean square deviation; and *SB* = structure-based probability.

Ligand	Docking Score (kJ/mol)	SD	RMSD	*SB*
**101**	−449.5	2.8	1.5	1.00
**270**	−437.6	7.4	1.6	0.97
**302**	−423.0	9.4	1.3	0.94
**299**	−422.7	9.2	1.3	0.94
**175**	−421.8	18.0	1.0	0.94
**298**	−420.2	20.1	1.6	0.93
**174**	−419.9	9.7	1.4	0.93
**173**	−419.7	7.4	1.3	0.93
**135**	−416.7	9.1	1.1	0.93
**MTX**	−560.4	17.6	0.4	-

**Table 2:** Kauranes classified as active using an approach combining ligand-based and structure-based VS.

Kaurane	*SB*	*LB*	*CA_Lm_*
**135**	0.93	0.57	0.70
**101**	1.00	0.51	0.69
**302**	0.94	0.54	0.68
**134**	0.90	0.55	0.68
**298**	0.93	0.53	0.68

**Table 3:** Results for VS-selected diterpenes as inhibitors of *Lm*PTR1.

Compound	135	302	301	302a	301a	PMA
**IC_50_ (µM)**	8.6	9.6	21.2	6.1	23.2	1.11
**Confidence** **Interval (95%)**	9.4–7.9	10.7–8.6	23.4–18.9	7.1–5.2	26.3–20.4	1.20–1.01
**Ki^app^**	1.88	2.10	4.64	1.33	5.08	0.24

**Table 4:** Kauranes classified as active using an approach combining ligand-based and structure-based VS.

Kaurane	*Lb SB*	*Lp SB*	*La SB*	*CA*
**302a**	0.87	1.00	1.00	0.96
**301a**	0.86	0.97	0.98	0.94
**175**	0.90	0.95	0.92	0.92
**69**	0.93	0.94	0.88	0.92
**135**	1.00	0.88	0.82	0.90
**134**	0.93	0.80	0.87	0.87
**302**	0.85	0.89	0.82	0.86

**Table 5:** VINA scores and interactions of structure **302**, PMA and DHB with amino acid residues of *Lp*PTR1, *Lb*PTR1, and *La*PTR1. Critical interactions are highlighted in bold font.

Protein	Ligand	VINA Score (kcal/mol)	Interacting Residues
***Lp*PTR1**	**Structure 302**	−9.73	*Van der Waals*: A14, G20, **L19**, N110, **S112**, Y114, M179, I180, Q186, P187, **Y194**, G225, **L226**, **S227**, L228, F229, Y283; *Carbon H-bond:* K198; A*lkyl*: R18, L19; *π-alkyl*: M183; *π-sigma:* L188*; π-anion:* **D181**.
	**PMA**	−7.92	*H-bond* N110, I180; *Van der Waals*: R18, **L19**, **S112**, M179, **Y194**, K198, G225, **L226**, **S227**, L228; *π-alkyl*: Y114, F229; *π-π T-shaped:* Y114*; π-anion:* **D181**.
	**DHB**	−8.33	*H-bond*: M179, D181, K198, G224; *Van der Waals:* L19, S112, Y194, P224, L226, S227, F229; *Carbon H-bond:* I180; *π-π T-shaped*: Y114 π-*anion:* **D181**.
***Lb*PTR1**	**Structure 302**	−11.1	*H-bond* G225; *Van der Waals:* K17, R18, N110, **S112**, Y114 I180, D181, L188, Y194, K198, **S227**, **L228**, F229, P230, Y241; *π-sigma*: M233, L226; A*lkyl*: L19
	**PMA**	−7.41	*H-bond:* R14, L19, N110; *Van der Waals:* G20, C21, A111, **S112**, **S227**, **L228**; *π-alkyl*: R18, Y194; *π-sigma*: Y114.
	**DHB**	−7.75	*H-bond:* L19, N110, P224; *Van der Waals:* A14, K17, R18, G20, C21, **S112**, I179, I180, D181, A182, Y194, **S227**, **L228**.
***La*PTR1**	**Structure 302**	−9.87	*H-bond*: A15, **K16**; *Van der Waals:* T12, G13, A14, R17, L18, H36, Y37, H38, R39, S40, N109, S111, S146, **Y193**, K197; *Alkyl*: **P223** *π-alkyl:* A110, L66.
	**PMA**	−7.19	*H-bond*: G224; *Van der Waals:* S111, M178, V179, A181, **Y193**, L228, M232; π-*alkyl*: **P223**; *Alkyl:* F113, L187, L225, Y240 *π-anion*: D180.
	**DHB**	−7.61	*H-bond:***K16**, R17, N109; *Van der Waals:* G13, G19, M178, V179, D180, A181, **Y193**, K197, P223, G224, L225; *π-alkyl:* R17, L18.

**Table 6:** Binding free energies (kJ/mol) from the MM/PBSA calculations for three of the best-ranked structures identified for *Lb*PTR1; DHB and PMA were used as reference ligands.

	135		302		302a		PMA		DHB	
Energy Contribution	kJ/mol	SD	kJ/mol	SD	kJ/mol	SD	kJ/mol	SD	kJ/mol	SD
**Van der Waals**	−210.7	6.0	−170.8	7.9	−208.6	7.6	−138.8	1.7	−121.3	3.0
**Electrostatic**	−2.9	1.5	−26.7	3.4	−9.7	3.0	−145.0	2.5	−194.6	10.3
**Polar solvation**	103.6	4.1	95.5	9.9	100.7	13.1	186.4	5.9	221.4	12.0
**SASA**	−22.7	0.5	−19.4	0.9	−20.6	0.4	−12.7	0.4	−12.9	0.3
**Binding energy**	−132.7	7.6	−121.4	6.1	−138.3	9.3	−110.0	4.2	−107.4	6.1

## Data Availability

The supplementary material can be accesses directly from investigators by email.

## Supplementary Materials

The following are available online. Figure S1: Ramachandran plots for hybrid models of *L. panamensis*, *L. braziliensis*, and *L. amazonensis*. Table S1: ChEMBL structures with pIC_50_ values used for random forest model construction. Table S2: *LB* probability for the entire diterpenes dataset obtained from random forest model. Table S3: Docking energies for 360 diterpenes from the structure-based VS for *Lm*PTR1. Table S4: Kauranes *CA* scores using an approach combining ligand-based and structure-based VS. Table S5: Vina scores for diterpene structures in the active site of *Lb*, *Lp*, and *La*. Table S6: Prediction of ADMET properties.

## Sample Availability

Samples of all compounds are available from the authors.
