# Supplementary material for: Identification of Kaurane-Type Diterpenes as Inhibitors of Leishmania Pteridine Reductase I
Source: Molecules. 2021 May 21;26(11):3076. doi: 10.3390/molecules26113076 (PMC8196580; doi:10.3390/molecules26113076)
Supplement: Supplementary file 1 [file molecules-26-03076-s001.zip › molecules-1218281-supplementary/molecules-1218281-figure.pdf]

molecules

MDPI

Article

Identification of Kaurane-Type Diterpenes as Inhibitors of Leishmania Pteridine Reductase I

Chonny Herrera-Acevedo [1,4], Areli Flores-Gaspar [2,*], Luciana Scotti [1], Francisco Jaime Bezerra Mendonça-Junior [3], Marcus Tullius Scotti [1,*] and Ericsson Coy-Barrera [2,4]

[1] Post-Graduate Program in Natural and Synthetic Bioactive Products, Federal University of Paraíba, João Pessoa, PB 58051-900, Brazil; chonny622@gmail.com (C.H-A.); luciana.scotti@gmail.com (L.S.); mtscotti@gmail.com (M.S)

[2] Departamento de Química, Facultad de Ciencias Básicas y Aplicadas, Universidad Militar Nueva Granada, Cajicá 250247, Colombia; areli.flores@unimilitar.edu.co (A.F-G.)

[3] Laboratory of Synthesis and Drug Delivery, State University of Paraíba, João Pessoa, Brazil; franciscojbmendonca@yahoo.com.br (F.M-J)

[4] Bioorganic Chemistry Laboratory, Facultad de Ciencias Básicas y Aplicadas, Universidad Militar Nueva Granada, Cajicá 250247, Colombia; ericsson.coy@unimilitar.edu.co (E.C-B.)

* Correspondence: mtscotti@gmail.com, Tel.: +55-83-99869-0415 (M.S); areli.flores@unimilitar.edu.co Tel: +57 (1)-650-00-00 ext. 1526. (A.F-G.)

Supplementary material

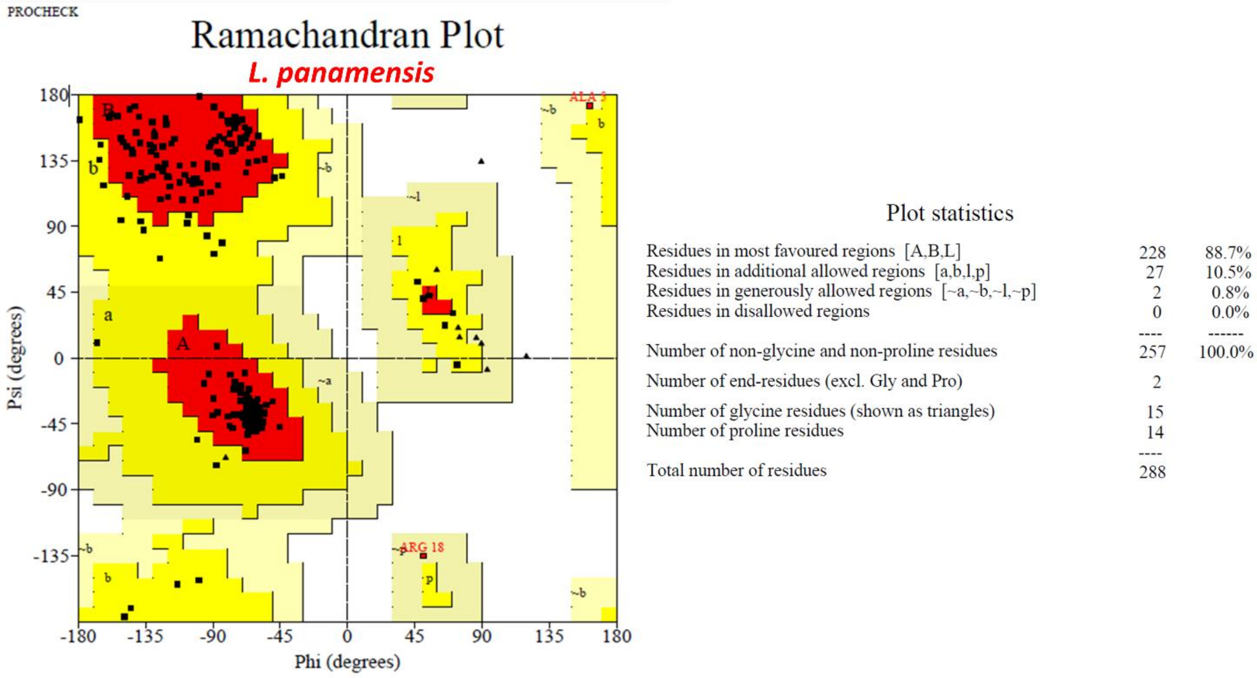

Plot statistics

Residues in most favoured regions [A,B,L]	228	88.7%
Residues in additional allowed regions [a,b,l,p]	27	10.5%
Residues in generously allowed regions [~a,~b,~l,~p]	2	0.8%
Residues in disallowed regions	0	0.0%
	----	------
Number of non-glycine and non-proline residues	257	100.0%
Number of end-residues (excl. Gly and Pro)	2	
Number of glycine residues (shown as triangles)	15	
Number of proline residues	14	

Total number of residues	288	

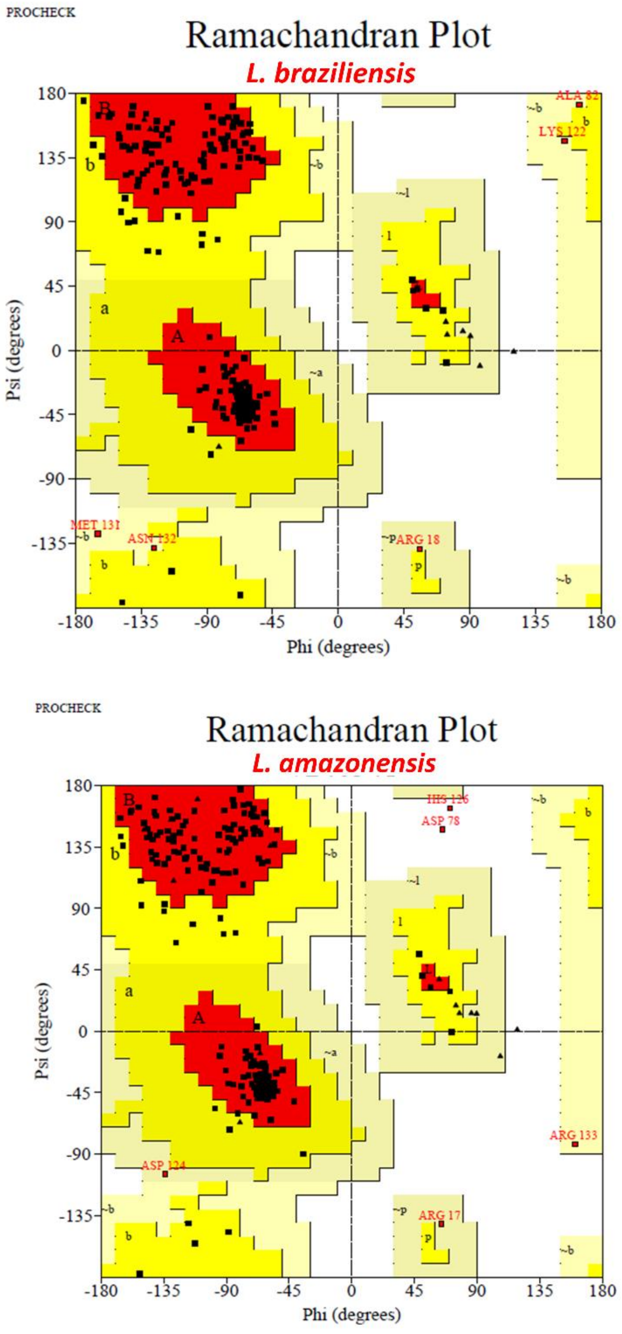

Plot statistics

Residues in most favoured regions [A,B,L]	232	89.9%
Residues in additional allowed regions [a,b,l,p]	21	8.1%
Residues in generously allowed regions [~a,~b,~l,~p]	5	1.9%
Residues in disallowed regions	0	0.0%
	----	------
Number of non-glycine and non-proline residues	258	100.0%
Number of end-residues (excl. Gly and Pro)	2	
Number of glycine residues (shown as triangles)	14	
Number of proline residues	14	

Total number of residues	288	

Plot statistics

Residues in most favoured regions [A,B,L]	222	88.8%
Residues in additional allowed regions [a,b,l,p]	23	9.2%
Residues in generously allowed regions [~a,~b,~l,~p]	3	1.2%
Residues in disallowed regions	2	0.8%
	----	------
Number of non-glycine and non-proline residues	250	100.0%
Number of end-residues (excl. Gly and Pro)	2	
Number of glycine residues (shown as triangles)	19	
Number of proline residues	16	

Total number of residues	287	

Figure S1. Ramachandran plots for hybrid models of *L. panamensis, L. braziliensis* and *L. amazonensis.*
